# An Empirical Study of Chronic Diseases in the United States: A Visual Analytics Approach to Public Health

**DOI:** 10.3390/ijerph15030431

**Published:** 2018-03-01

**Authors:** Wullianallur Raghupathi, Viju Raghupathi

**Affiliations:** 1 Gabelli School of Business, Fordham University, New York, NY 10023, USA; Raghupathi@fordham.edu; 2 Koppelman School of Business, Brooklyn College of the City University of New York, Brooklyn, NY 11210, USA

**Keywords:** behavioral health, chronic disease, comorbidity, overarching condition, population health, preventive health

## Abstract

In this research we explore the current state of chronic diseases in the United States, using data from the Centers for Disease Control and Prevention and applying visualization and descriptive analytics techniques. Five main categories of variables are studied, namely chronic disease conditions, behavioral health, mental health, demographics, and overarching conditions. These are analyzed in the context of regions and states within the U.S. to discover possible correlations between variables in several categories. There are widespread variations in the prevalence of diverse chronic diseases, the number of hospitalizations for specific diseases, and the diagnosis and mortality rates for different states. Identifying such correlations is fundamental to developing insights that will help in the creation of targeted management, mitigation, and preventive policies, ultimately minimizing the risks and costs of chronic diseases. As the population ages and individuals suffer from multiple conditions, or comorbidity, it is imperative that the various stakeholders, including the government, non-governmental organizations (NGOs), policy makers, health providers, and society as a whole, address these adverse effects in a timely and efficient manner.

## 1. Introduction

A chronic condition “is a physical or mental health condition that lasts more than one year and causes functional restrictions or requires ongoing monitoring or treatment” [1,2]. Chronic diseases are among the most prevalent and costly health conditions in the United States. Nearly half (approximately 45%, or 133 million) of all Americans suffer from at least one chronic disease [3,4,5], and the number is growing. Chronic diseases—including, cancer, diabetes, hypertension, stroke, heart disease, respiratory diseases, arthritis, obesity, and oral diseases—can lead to hospitalization, long-term disability, reduced quality of life, and death [6,7]. In fact, persistent conditions are the nation’s leading cause of death and disability [6].

Globally, chronic diseases have affected the health and quality of life of many citizens [8,9]. In addition, chronic diseases have been a major driver of health care costs while also impacting workforce patterns, including, of course, absenteeism. According to the Centers for Disease Control, in the U.S. alone, chronic diseases account for nearly 75 percent of aggregate healthcare spending, or an estimated $5300 per person annually. In terms of public insurance, treatment of chronic diseases comprises an even larger proportion of spending: 96 cents per dollar for Medicare and 83 cents per dollar for Medicaid [4,10,11,12]. Thus, the understanding, management, and prevention of chronic diseases are important objectives if, as a society, we are to provide better quality healthcare to citizens and improve their overall quality of life.

More than two thirds of all deaths are caused by one or more of these five chronic diseases: heart disease, cancer, stroke, chronic obstructive pulmonary disease, and diabetes. Additional statistics are quite stark [5,13]: chronic diseases are responsible for seven out of 10 deaths in the U.S., killing more than 1.7 million Americans each year; and more than 75% of the $2 trillion spent on public and private healthcare in 2005 went toward chronic diseases [5]. What makes treating chronic conditions (and efforts to manage population health) particularly challenging is that chronic conditions often do not exist in isolation. In fact, today one in four U.S. adults have two or more chronic conditions [5], while more than half of older adults have three or more chronic conditions. And the likelihood of these types of comorbidities occurring goes up as we age [5]. Given America’s current demographics, wherein 10,000 Americans will turn 65 each day from now through the end of 2029 [5], it is reasonable to expect that the overall number of patients with comorbidities will increase greatly.

Trends show an overall increase in chronic diseases. Currently, the top ten health problems in America (not all of them chronic) are heart disease, cancer, stroke, respiratory disease, injuries, diabetes, Alzheimer’s disease, influenza and pneumonia, kidney disease, and septicemia [14,15,16,17,18]. The nation’s aging population, coupled with existing risk factors (tobacco use, poor nutrition, lack of physical activity) and medical advances that extend longevity (if not also improve overall health), have led to the conclusion that these problems are only going to magnify if not effectively addressed now [19].

A recent Milken Institute analysis determined that treatment of the seven most common chronic diseases coupled with productivity losses will cost the U.S. economy more than $1 trillion dollars annually. Furthermore, compared with other developed nations, the U.S. has ranked poorly on cost and outcomes. This is predominantly because of our inability to effectively manage chronic disease. And yet the same Milken analysis estimates that modest reductions in unhealthy behaviors could prevent or delay 40 million cases of chronic illness per year [11]. If we learn how to effectively manage chronic conditions, thus avoiding hospitalizations and serious complications, the healthcare system can improve quality of life for patients and greatly reduce the ballooning cost burden we all share [10].

The success of population health and chronic disease management efforts hinges on a few key elements: identifying those at risk, having access to the right data about this population, creating actionable insights about patients, and coaching them toward healthier choices. Methods such as data-driven visual analytics help experts analyze large amounts of data and gain insights for making informed decisions regarding chronic diseases [10,20]. According to the U.S.-based Institute of Medicine and the National Research, the vision for 21st century healthcare includes increased attention to cognitive support in decision making [21]. This encompasses computer-based tools and techniques that aid comprehension and cognition. Visualization techniques offer cognitive support by offering mental models of the information through a visual interface [22]. They combine statistical methods and models with advanced interactive visualization methods to help mask the underlying complexity of large health data sets and make evidence-based decisions [23]. Chronic diseases are characterized by high prevalence among populations, rising complication rates, and increased incidence of people with multiple chronic conditions, to name a few. In this scenario, visualization can represent association between preventive measures and disease control, summary health dimensions across diverse patient populations and, timeline of disease prevalence across regions/populations, to offer actionable insights for effective population management and national development [24]. Additionally, visual techniques offer the ability to analyze data at multiple levels and dimensions starting from population to subpopulation to the individual [25]. This paper addresses the challenge of understanding large amounts of data related to chronic diseases by applying visual analytics techniques and producing descriptive analytics. Our overall goal is to gain insight into the data and make policy recommendations.

Given that large segments of the U.S. population suffer from one or more chronic disease conditions, a data-driven approach to the analysis of the data has the potential to reveal patterns of association, correlation, and causality. We therefore studied the variables extracted from a highly reliable source, the Centers for Disease Control. Data for variables pertaining to several categories, namely chronic condition (“condition” is used interchangeably with “disease”), behavioral health, mental health, preventive health, demographics, overarching conditions, and location for several years (typically 2012 to 2014). We analyzed relationships within each category and across categories to obtain multi-dimensional views and insight into the data. The analytics provide insights and implications that suggest ways for the healthcare system to better manage population health.

This paper is organized as follows: Section 1 offers an introduction to the research, Section 2 discusses the methodology, Section 3 presents and discusses the visual charts and results, Section 4 contains the scope and limitations of the research, Section 5 describes the policy implications and future research, and Section 6 presents our conclusions.

## 2. Materials and Methods 

This study analyzes the characteristics of chronic diseases in the U.S. and explores the relationships between demographics, behavior habits, and other health conditions and chronic diseases, thereby revealing information for public health practice at the state-specific level. In this data-driven study we use visual analytics [26], conducting primarily descriptive analytics [20] to obtain a panoramic insight into the chronic diseases data set pulled from the Centers for Disease Control and Prevention web site. The discipline of visual analytics aims to provide researchers and policymakers with better and more effective ways to understand and analyze large data sets, while also enabling them to act upon their findings in real time. Visual analytics integrates the analytic capabilities of the computer and the abilities of human analysts, thus inviting novel discoveries and empowering individuals to take control of the analytical process. It sheds light on unexpected and hidden insights, which may lead to beneficial and profitable innovation [27,28]. Driving visual analytics is the aim of turning information overload into opportunity; just as information visualization has changed our view on databases, the goal of visual analytics is to make our way of processing data and information transparent and accessible for analytic discourse. The visualization of these processes provides the means for examining the actual processes and not just the results. Visual analytics applies such technology as business intelligence (BI) tools to combine human analytical skill with computing power. Clearly, this research is highly interdisciplinary, involving such areas as visualization, data mining, data management, data fusion, statistics, and cognitive science, among others. One key understanding of visual analytics is that the integration of these diverse areas is a scientific discipline in its own right [29,30].

Historically, automatic analysis techniques, such as statistics and data mining, were developed independently of visualization and interaction techniques. One of the most important steps in the direction of visual analytics research was the need to move from confirmatory data analysis (using charts and other visual representations to present results) to exploratory data analysis (interacting with the data), first introduced to the statistics research community by John W. Tukey in his book, *Exploratory Data Analysis* [31].

With improvements in graphical user interfaces and interaction devices, the research community devoted its efforts to information visualization [27]. Eventually, this community recognized the potential of integrating the user’s perspective into the knowledge discovery and data mining process through effective and efficient visualization techniques, interaction capabilities, and knowledge transfer. This led to visual data exploration and visual data mining [29] and widened considerably the scope of applications of visualization, statistics, and data mining—the three pillars of analytics. In visual analytics is defined as “the science of analytical reasoning facilitated by interactive human-machine interfaces” [29]. A more current definition says “visual analytics combines automated analysis techniques with interactive visualizations for an effective understanding reasoning and decision-making on the basis of very large and complex data sets” (both reported in [27]). In their book *Illuminating the Path*, Thomas and Cook define visual analytics as the science of analytical reasoning facilitated by interactive visual interfaces.

One application of visualization is descriptive analytics, the most commonly used and most well understood type of analytics. It was the earliest to be introduced and the easiest by far to implement and understand in that it describes data “as is” without complex calculations. Descriptive analytics is more data-driven than other models. Most health data analyses start with descriptive analytics, using data to understand past and current health patterns and trends and to make informed decisions [20]. The models in descriptive analytics categorize, characterize, aggregate, and classify data, converting it into information for understanding and analyzing business decisions, outcomes, and quality. Such data summaries can be in the form of meaningful charts and reports, and responses to queries using SQL. Descriptive analytics uses a significant amount of visualization. One could, for example, obtain standard and customized reports and drill down into the data, running queries to better understand, say, the sales of a product [20]. Descriptive analytics helps answer such questions as: How many patients with diabetes also have obesity? Which of the chronic diseases are more prevalent in different regions of the country? What behavioral habits are correlated to the chronic diseases? Which groups of patients suffer from more than one chronic condition? Is there an association between health insurance (and lack thereof) and chronic diseases? What are cost trade-offs between chronic disease prevention and management? What are typical patient profiles for various chronic diseases? 

This study concentrates on chronic condition indicators and related demographics, behavior habits, preventive health, and oral health factors. As mentioned, the data source for this study is the Center for Disease Control and Prevention (CDC) [32]. The CDC’s Division of Population Health offers a crosscutting set of 124 indicators that were developed by consensus. Those indicators are integrated from multiple resources, with the help of the Chronic Disease Indicator web site, which serves as a gateway to additional information and data sources. In this research we downloaded secondary data for the United States from the CDC dataset, for the years 2012 to 2014. The data is for states, territories, and large metropolitan areas in the U.S., including the 50 states and District of Columbia, Guam, Puerto Rico, and the U.S. Virgin Islands. Data cleaning, integration, and transformation were conducted on the raw data set. The main categories of variables included—chronic condition, mental health, behavior habits, preventative health, and demographics. In addition, overarching conditions and location were also studied. Table 1 summarizes the categories and variables.

## 3. Results

We use visualization and descriptive analytics to explore chronic conditions, preventive healthcare, mental health, and overarching conditions, with the objective of deciphering relationships and patterns that emerge from the visualization. We would like to point out that since our sample includes adults aged 18 and over our results are applicable for adults in that age group.

**Figure 1:**
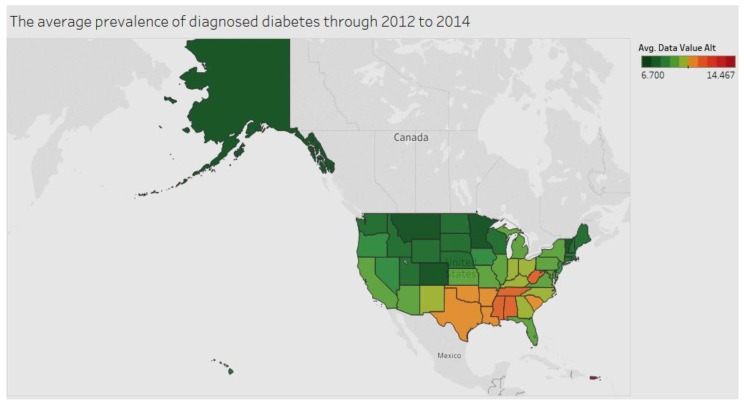
Diabetes by state.

Figure 1 models the average prevalence of diagnosed diabetes among adults aged ≥18 years in the period 2012 to 2014. Puerto Rico leads the pack, followed by Mississippi.

**Figure 2:**
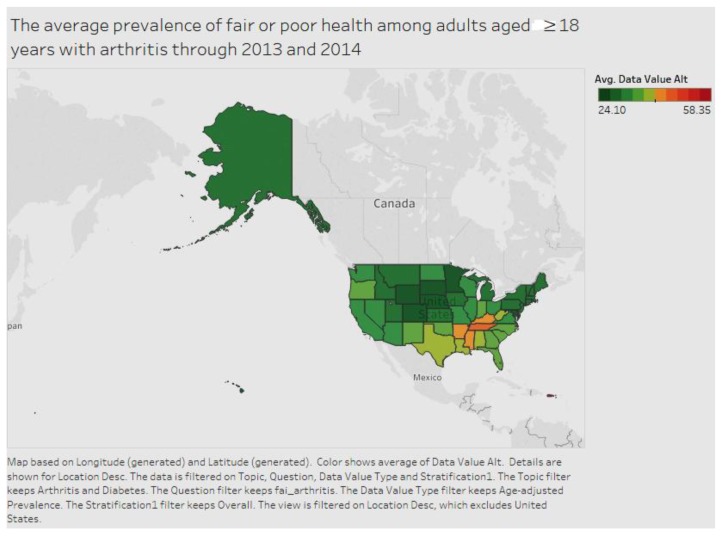
Arthritis by state.

As Figure 2 below shows, Puerto Rico has the highest number of citizens among adults aged ≥18 years, in fair or poor health with arthritis for the period 2013 to 2014. Puerto Rico is followed by Tennessee and Mississippi.

**Figure 3:**
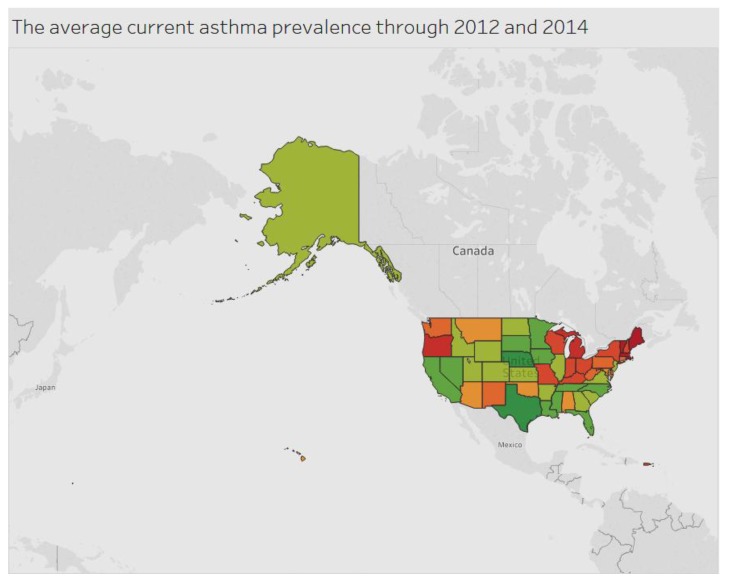
Asthma by state.

The current asthma prevalence among adults aged ≥18 years for the period 2012 to 2014 is indicated in Figure 3. West Virginia has a higher prevalence of the condition compared to other states.

**Figure 4:**
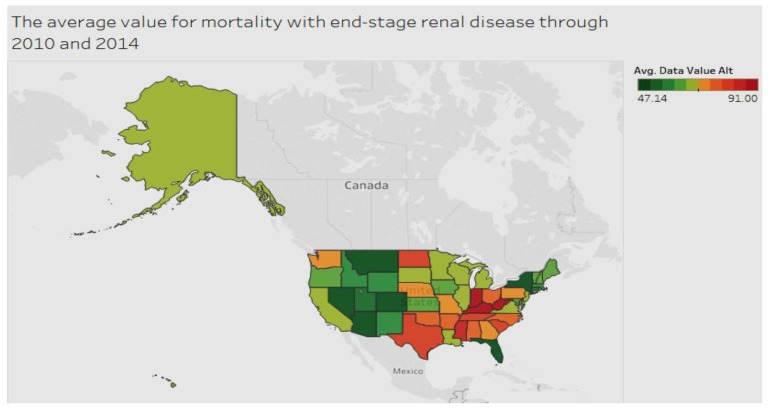
End-stage renal disease by region.

With regard to end-stage renal disease, Figure 4 shows that the condition is dispersed widely among various areas.

**Figure 5:**
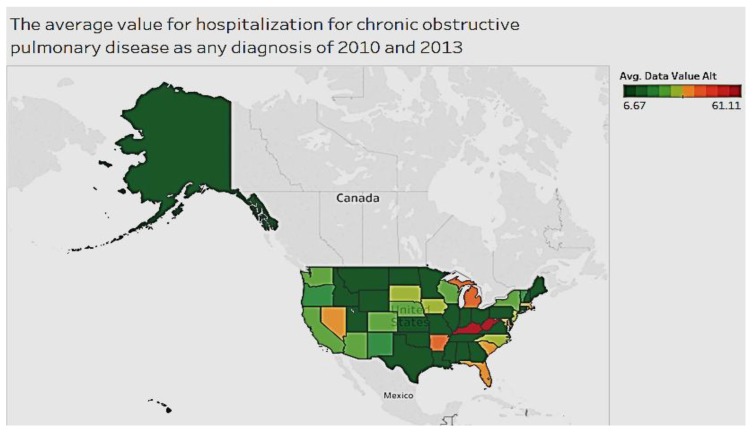
Chronic obstructive pulmonary disease by state.

The average value for hospitalization for chronic obstructive pulmonary disease for all diagnoses between 2010 and 2013 is shown in Figure 5. Kentucky and West Virginia have higher hospitalizations compared to other states. Most of the areas are below 45 cases per 100,000.

In exploring chronic conditions by location in the U.S., we see that some conditions, such as diabetes, arthritis, and obstructive pulmonary diseases, are more prevalent in eastern states, while others, such as asthma, occur more often in northeastern states. For diabetes, listed as a cause of death for the years 2010 to 2014, the states of Oklahoma and West Virginia had the relatively high average threshold of over 100 (age adjusted rate per 100,000). In the case of asthma, West Virginia has the highest prevalence of the condition (among adults), while Maryland, Massachusetts, and New York had the highest number of hospitalizations. With regard to chronic obstructive pulmonary disease, Kentucky and West Virginia had the most hospitalizations compared to other states. The majority of states are indeed below 45 cases per 100,000. With respect to arthritis among adults, a majority of states average below 25%, with the exception of West Virginia, which averaged 34.15%. In summary, West Virginia ranks high in prevalence for most chronic conditions, such as diabetes, asthma, chronic pulmonary disease, and arthritis when compared to all other states for the period 2000 to 2014.

**Figure 6:**
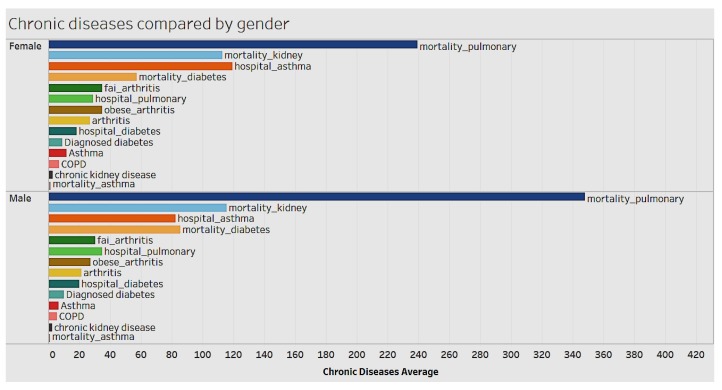
Chronic condition by gender.

**Figure 7:**
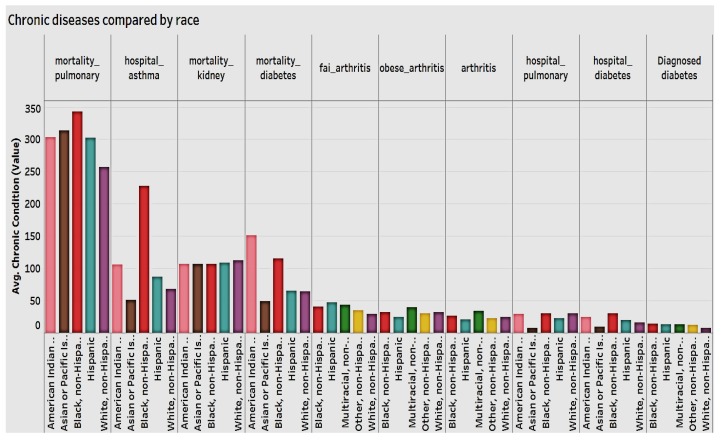
Chronic conditions by race.

We looked at the distribution of chronic conditions by gender and race to identify relevant trends and patterns (Figure 6 and Figure 7). Chronic conditions differ by gender. Women tend to have significantly higher cases per 100,000 of hospitalizations for asthma. Whereas men tend to have a higher mortality rate from chronic obstructive pulmonary disease, diabetes, chronic kidney, and other conditions, as shown in Figure 6.

We also examined all chronic conditions by race (Figure 7) and found that non-Hispanic Blacks have higher mortality rate for pulmonary disease and asthma and a higher hospitalization from diabetes. They are followed by Pacific Islander and American Indians. All categories of arthritis are fairly evenly distributed among Black, non-Hispanic, Multiracial, Whites, and other.

Females have a higher hospitalization rate for asthma (per 100,000), while in terms of mortality rate for chronic obstructive pulmonary disease, diabetes, and chronic kidney disease, males have the higher hospitalization rate. Again, American Indian or Alaskan Natives have higher mortality rate for chronic obstructive pulmonary disease, diabetes, and kidney disease. They’re followed by Blacks and non-Hispanics.

### 3.1. Mental Health by Gender and Race

**Figure 8:**
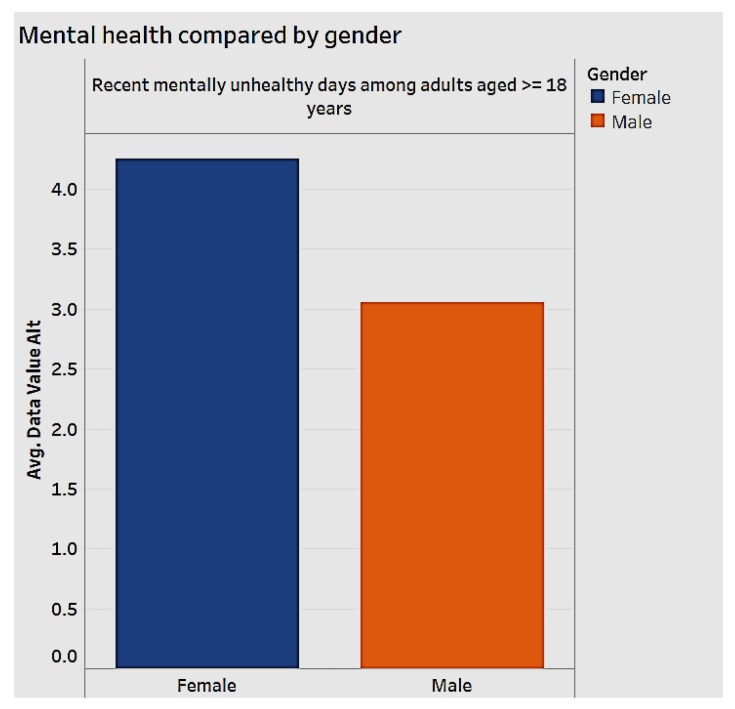
Mental health by gender.

**Figure 9:**
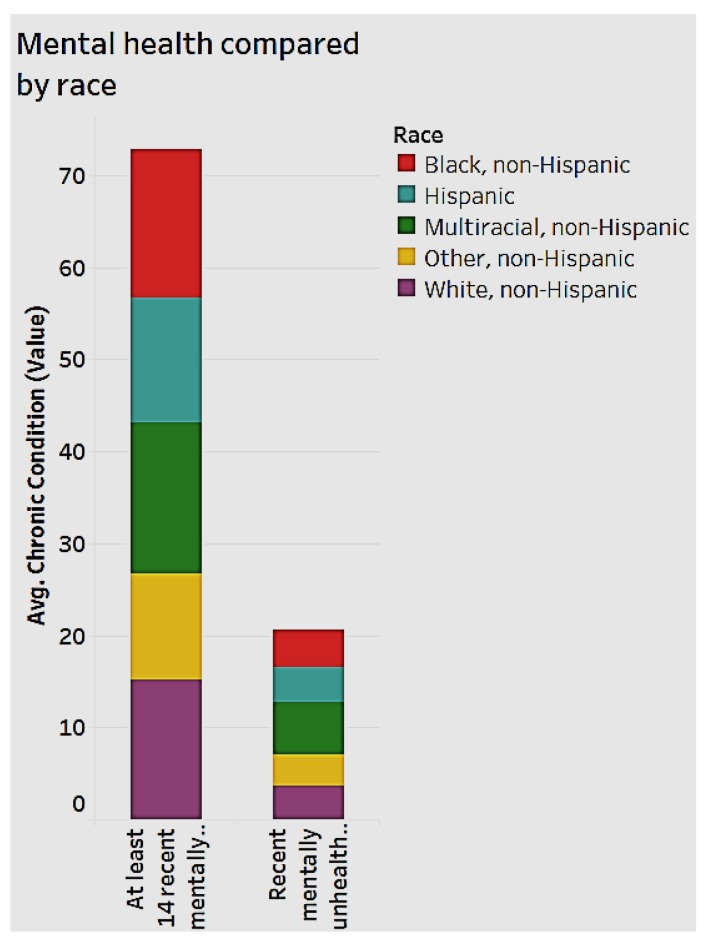
Mental health by race.

Mental health is an important aspect of national healthcare impacting chronic diseases. We analyzed mental health by gender (Figure 8) and by race (Figure 9). When we examine how many days an individual feels “mentally unhealthy" for the years 2012 to 2014, women are more likely to have more unhealthy days than men, as shown in Figure 8.

Simultaneously, multi-racial, non-Hispanic women in the age group 18 to 44 have a higher crude prevalence rate of at least 14 recent “mentally unhealthy” days. This group is followed by black non-Hispanics.

We then studied behavioral habits in the data set to gain insight into noticeable patterns, if in fact any exist.

### 3.2. Behavioral Habits by Gender and Race

**Figure 10:**
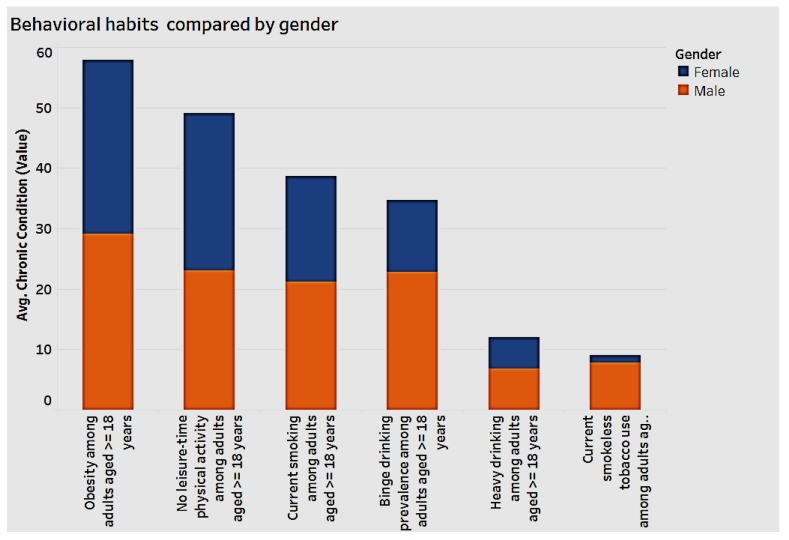
Behavioral habits by gender.

Figure 10 charts behavioral habits by gender.

As seen in the chart above, men display higher numbers in the alcohol categories of “binge drinking” and “heavy drinking”, as well as in “current smokeless” tobacco use among adults. In terms of engaging in “current smoking”, “obesity”, and “no leisure-time” physical activity, both men and women experience similar complications, that highlights the need for positive behavior modification.

**Figure 11:**
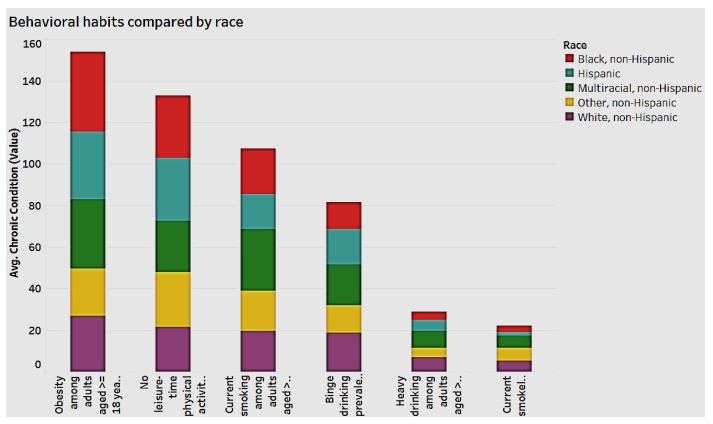
Behavioral habits by race.

Figure 11 illustrates the analysis of behavioral habits by race.

Figure 11 reveals that for the behavioral habits of “obesity” and “no leisure-time” physical activity among adults aged 18 and over, the black non-Hispanic and Hispanic races have the highest frequency, while white non-Hispanics have the lowest. By and large, in most behavioral habits, the other non-Hispanics have the lowest frequency.

### 3.3. Preventive Health and Chronic Conditions

**Figure 12:**
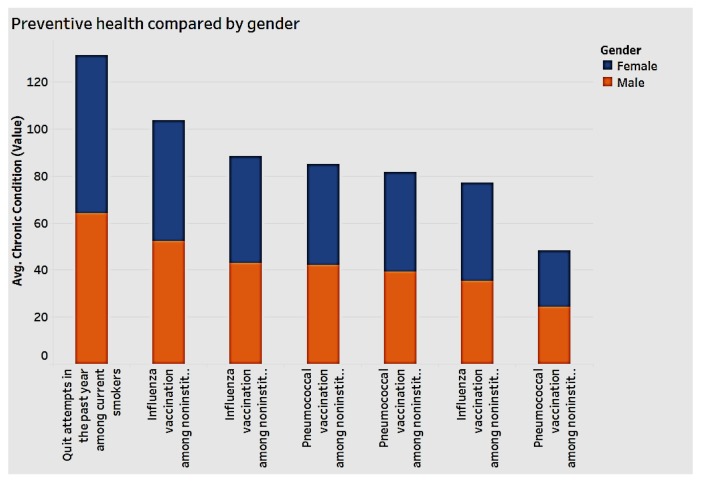
Preventive health by gender.

**Figure 13:**
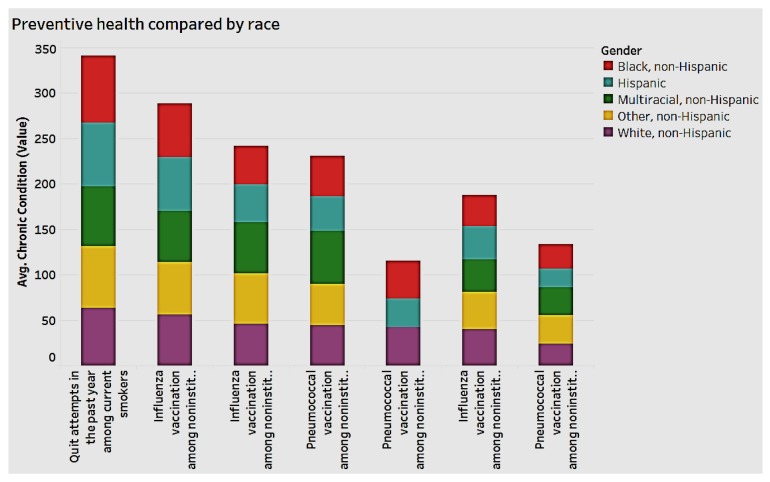
Preventive health by race.

We analyzed the data to detect associations between demographics and preventive health. As Figure 12 indicates, both men and women appear to engage in preventive health, though women have the edge. With regard to race, Blacks and Hispanics engage less in preventive health overall, as shown in Figure 13.

**Figure 14:**
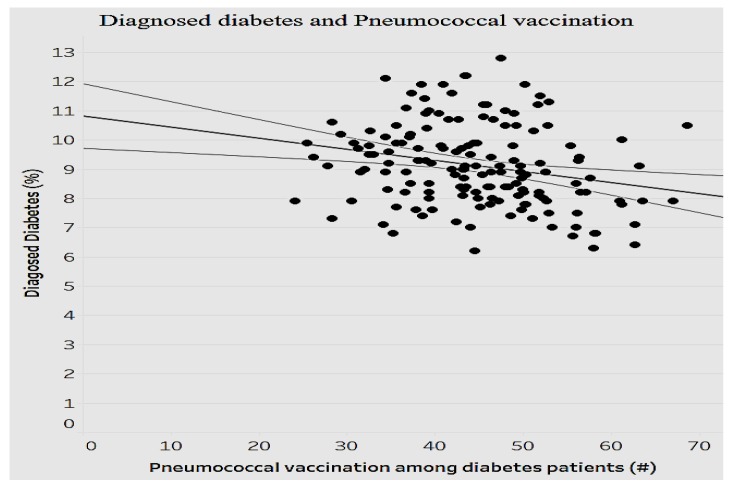
Diagnosed diabetes ratio by pneumococcal vaccination ratio. (#: number).

While all chronic conditions are debilitating on the economy, for the sake of scope, we selectively analyze the influence of a few conditions such as diabetes and asthma. By 2034, the population with diabetes is expected to increase by 100% and the cost expected to increase by 53% [33]. Figure 14 depicts the association between diabetes and pneumococcal vaccination for diabetes.

**Figure 15:**
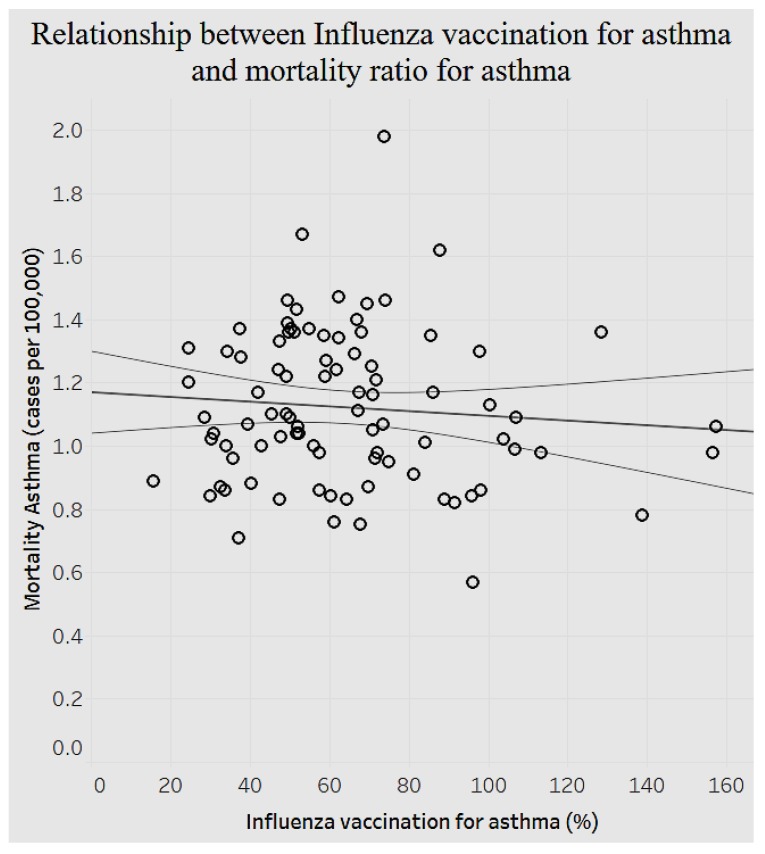
Mortality ratio for asthma and influenza vaccination for asthma.

As indicated in Figure 14, there is a significant negative relationship between the average pneumococcal vaccination among diabetes patients and the average diagnosed diabetes ratio among the population (*p* < 0.0001). As the average pneumococcal vaccination among diabetes patients increases, the average diagnosed diabetes ratio decreases (fewer cases of diabetes). Given the importance of asthma as another prevalent chronic condition, we decided to analyze the relationship between the mortality ratio and influenza vaccinations for asthma to determine the efficiency of preventive measures (Figure 15).

Figure 15 shows a significant negative association (*p* < 0.0001): as the rate of influenza vaccination for asthma increases, the mortality ratio of asthma declines. Analysis of the above preventive health variables shows that resources and efforts dedicated to preventive healthcare offer promise. The importance of managing chronic diseases is also highlighted when we examine the association between behavioral habits and overarching conditions.

### 3.4. Behavioral Health and Overarching Conditions

Overarching conditions represent situations or factors that directly or indirectly influence the area of study. In our research we look at the influence of these conditions on chronic diseases, behavioral health, and preventive health. The overarching conditions include lack of health insurance (%), self-rated health status (good, fair, poor), and prevalence of sufficient sleep (%) for which data was available.

**Figure 16:**
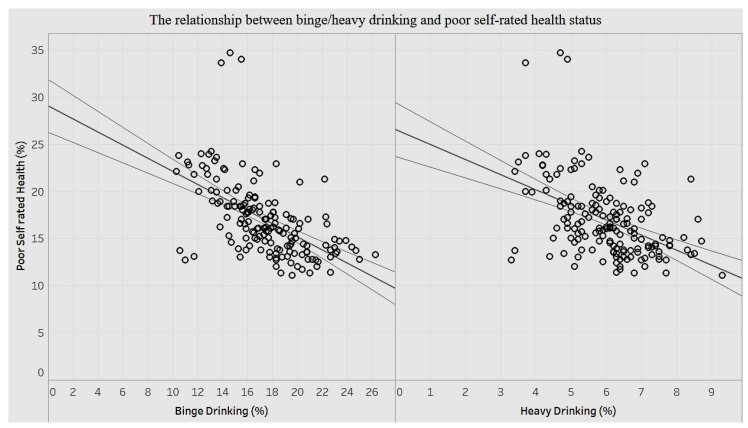
Binge/heavy drink and poor self-rated health status.

We explored the association of self-assessed health statuses among adults with the behavioral habits of binge drinking and heavy drinking (Figure 16).

There is a significant negative correlation (*p* < 0.0001) between binge drinking and self-assessment of health. That is to say that the lower the health self-assessment, the higher is the percentage of binge drinking. A decrease of less than 1% (0.69%) in self-assessed health is associated with a 1% increase in binge drinking. Likewise, there is a significant negative association (*p* < 0.0001) between self-assessment of health and percentage of heavy drinking. A decrease of 1.6% in self-assessed health is associated with a 1% increase in heavy drinking. We can surmise that reduced self-assessment of health has a stronger influence on heavy drinking than binge drinking among adults.

**Figure 17:**
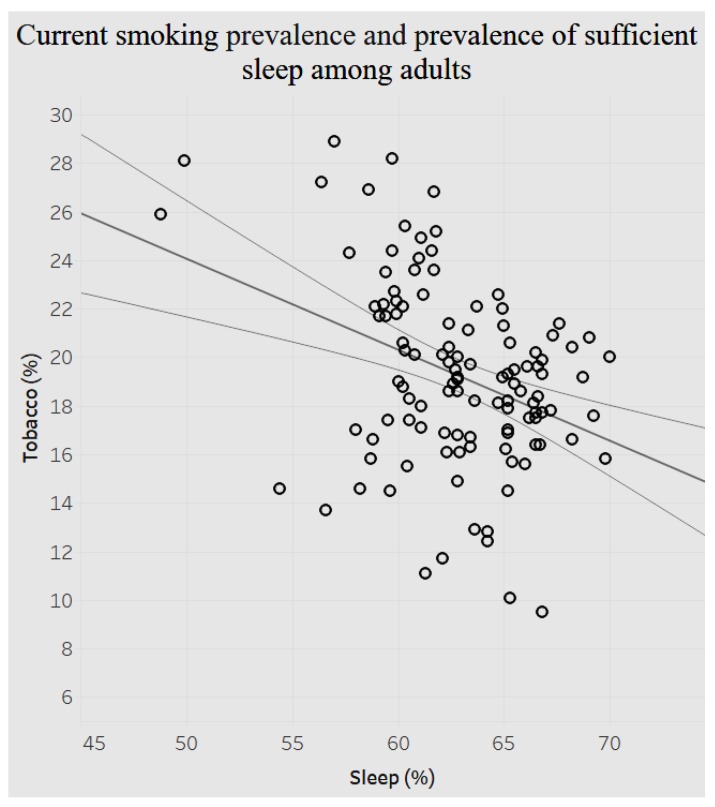
Current smoking prevalence by presence of sufficient sleep among adults.

Next, we looked at the association between current smoking prevalence and presence of sufficient sleep among adults (Figure 17).

Figure 17 above shows a significant negative association (*p* < 0.0001) between prevalence of current smoking and prevalence of sufficient sleep. When current smoking prevalence decreases by less than 1% (0.38%), the prevalence of sufficient sleep increases by 1%.

**Figure 18:**
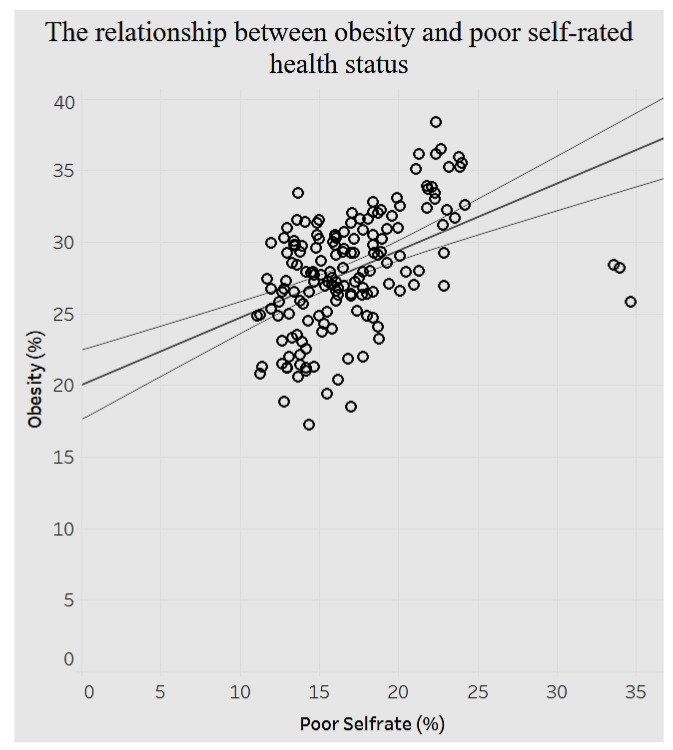
Obesity by poor self-rated health status.

The relationship between poor self-rated health status and obesity is positive (Figure 18). The higher the prevalence of fair or poor self-rated health, the higher is the prevalence of obesity. When poor self-rated health increases by 1%, the prevalence of obesity increases by 0.468779%.

**Figure 19:**
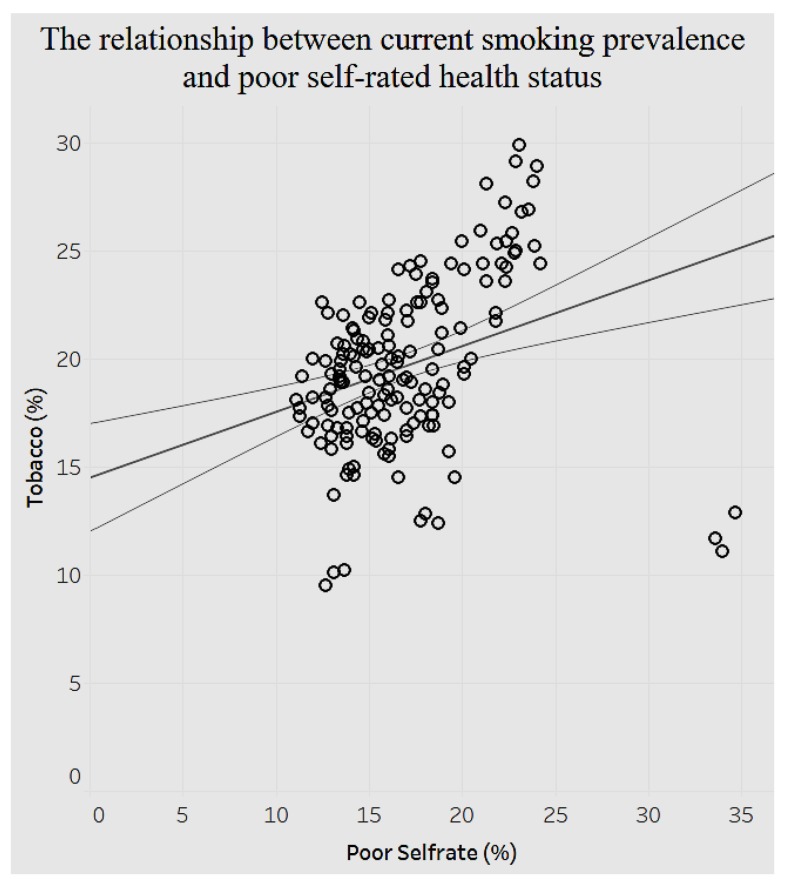
Smoking by self-rated health status.

Similarly, poor self-rated health has a positive association with current smoking, as indicated in Figure 19. As the prevalence of poor self-rated health increases by 1%, the prevalence of current smoking increases by 0.30425%.

### 3.5. Chronic Conditions and Overarching Conditions

**Figure 20:**
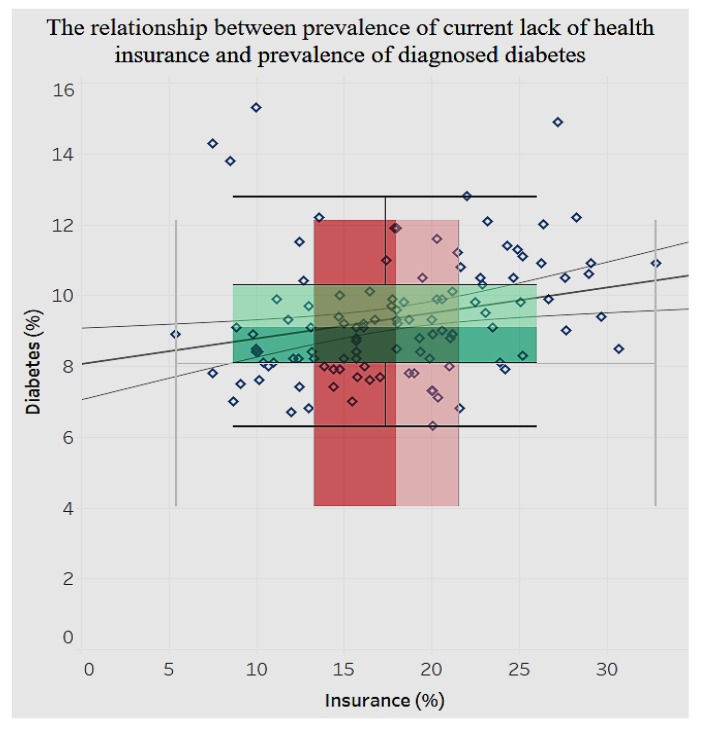
Current lack of health insurance by diagnosed diabetes.

In the analysis of various chronic conditions, there are significant clusters of conditions among men and women, such as the prevalence of asthma, with the women tending to have a higher prevalence of asthma than men. Regarding such chronic conditions as diabetes, there is a significant positive relationship (*p* < 0.001) between lack of health insurance and prevalence of diagnosed diabetes (Figure 20).

**Figure 21:**
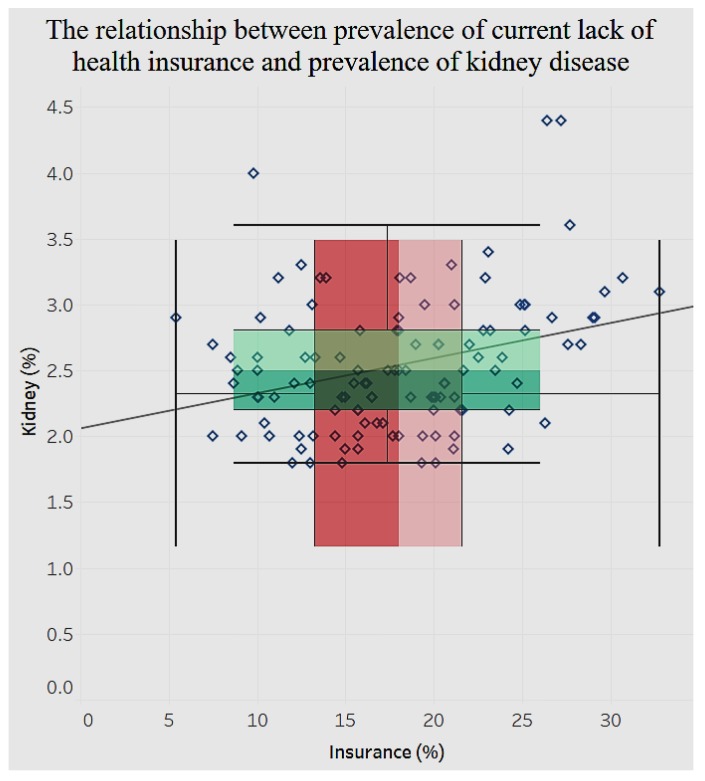
Lack of insurance by chronic kidney disease.

We notice in Figure 20 that the distribution of lack of health insurance is sparse compared to that of diagnosed diabetes among adults aged 18 and older. Likewise, for chronic kidney disease (Figure 21) there is a significant positive relationship (*p* < 0.0001) with lack of health insurance.

**Figure 22:**
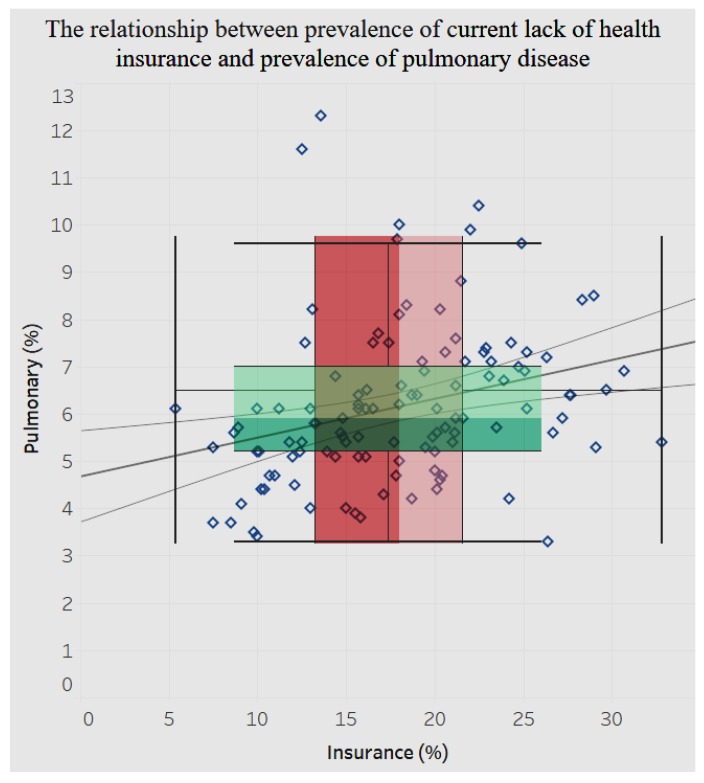
Lack of insurance by pulmonary disease.

The relationship between lack of insurance and hospitalization for chronic pulmonary disease is positive and significant (*p* < 0.0001), as shown in Figure 22. An increase in the lack of insurance is associated with an increase in hospitalization for chronic pulmonary disease.

### 3.6. Association between Chronic Conditions

**Figure 23:**
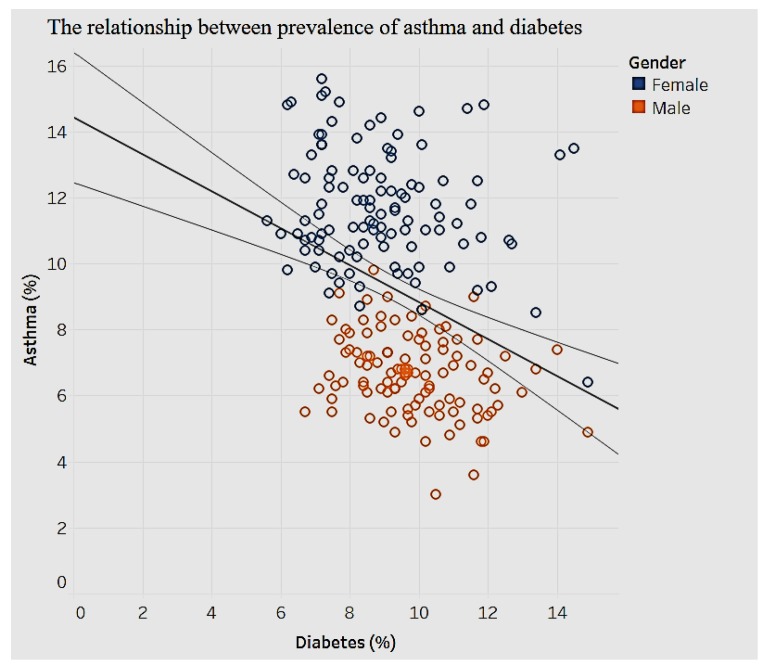
Asthma by diabetes.

We analyzed for any associations between different chronic conditions. It is important to incorporate gender as a factor in the association and prevalence of chronic diseases, so as to develop customized plans for diagnoses and treatments. A linear trend model was developed for the relationship between asthma and diabetes (Figure 23).

The model in Figure 23 shows a significant negative relationship (*p* < 0.01) between asthma and diabetes. We can see gender clusters for the prevalence of asthma. Women tend to have higher prevalence of asthma compared to men. Overall, prevalence of asthma is negatively related to the prevalence of diabetes. On average, a high prevalence of asthma is associated with a low prevalence of diabetes. In terms of gender differences our results are consistent with other studies that have shown that women are more prone to develop asthma. Contributing factors include puberty, menstruation, pregnancy, menopause, and oral contraceptives [34,35]. There is potential for more research in this area.

**Figure 24:**
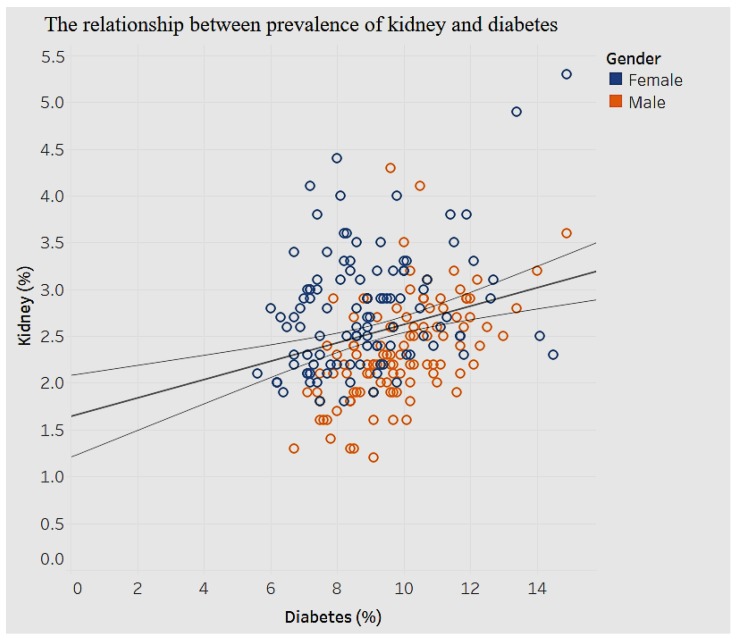
Diabetes by kidney disease.

The association between diabetes and kidney disease is shown in Figure 24.

Figure 24 shows a moderate, positive association (*p* < 0.01) between prevalence of kidney disease and diabetes. As the prevalence of diagnosed diabetes increases by 1%, the prevalence of chronic kidney disease increases by 0.09%. There are no obvious differences in gender here.

**Figure 25:**
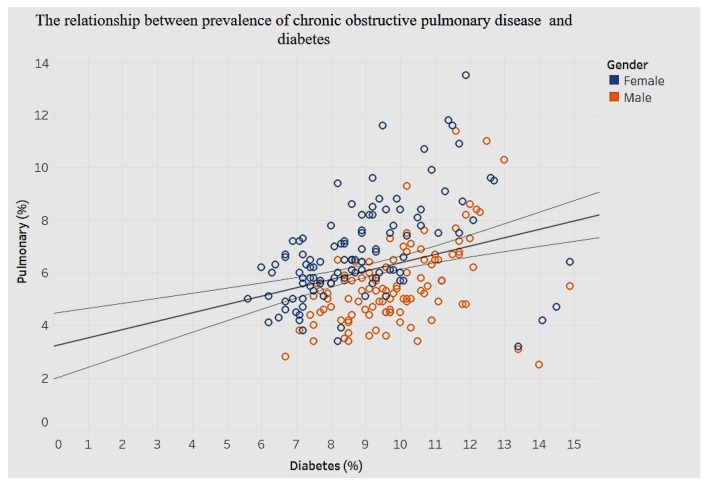
Diabetes by obstructive pulmonary disease.

**Figure 26:**
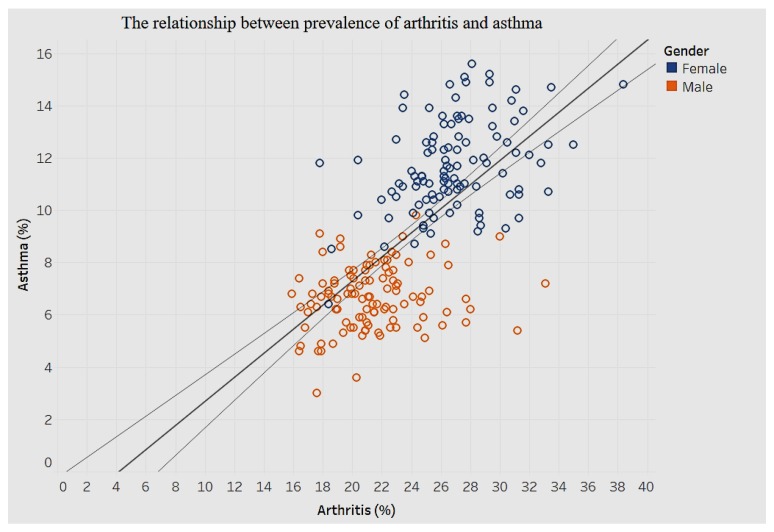
Arthritis by asthma.

The association between diabetes and chronic pulmonary disease is shown in Figure 25, and that between arthritis and asthma is shown in Figure 26.

In Figure 25, we find a significant positive association between diabetes and chronic pulmonary disease (*p* < 0.001).

When it comes to prevalence of arthritis and asthma, there clearly are clusters for men and women, as shown in Figure 26. There is a positive association such that an increase of 1% in prevalence of arthritis is associated with a 0.4% increase in prevalence of asthma.

**Figure 27:**
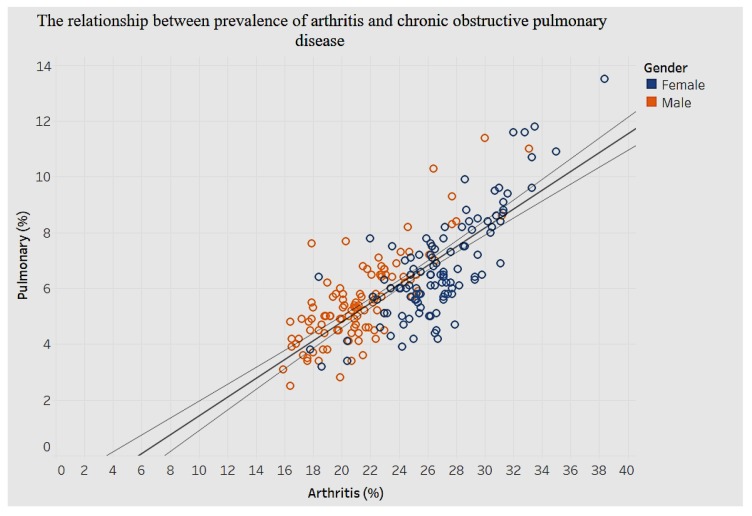
Arthritis by chronic obstructive pulmonary disease.

Figure 27 shows the association between arthritis and chronic pulmonary disease.

Although there are no defined clusters for men and women with regard to the prevalence of arthritis and chronic obstructive pulmonary disease, there is a significant positive association (*p* < 0.0001), as Figure 27 illustrates. An increase of 1% in prevalence of arthritis is associated with an increase of 0.3% in chronic obstructive pulmonary disease.

### 3.7. Summary of Results

The visual analytics figures above offer insight into a representative cross section of the data. They provide a bird’s eye view of the dimensions and correlations of chronic diseases “conditions”, behavioral health, and preventive health condition in the U.S. In addition, associations between mental health and chronic conditions, preventive health and chronic conditions, and among chronic conditions themselves highlight the dynamics of interplay between these categories. This understanding is useful to policymakers in framing appropriate health policies. Preventive healthcare and mental health are both important elements in the management, mitigation, and prevention of chronic conditions. By exploring these in the context of chronic conditions, we offer insight on allocation and prioritization of resources in mitigation and prospective eradication of chronic diseases at a national level. Overarching conditions, including a lack of health insurance, influence the access to necessary health services, including preventive care. This lack of availability is associated with poor health and the prevalence of chronic diseases. Similarly, self-assessed health status is a good indicator of overall health status, correlating with subsequent health service use, functional status, and mortality [36]. Poor mental health interferes with social functioning as well as health condition and should therefore be monitored in chronic disease mitigation. Experiencing activity limitation due to poor physical or mental health undermines efforts to achieve a healthy lifestyle and therefore should be addressed at individual, state, and national levels.

## 4. Scope and Limitations

Our research has a few limitations. First, our study is cross-sectional and covers only the years 2012 to 2014, the years for which data is available. Second, we included only a limited set of variables (indicators) from the large data repository on the CDC website. A more comprehensive study could draw from other sources and a larger set of variables. Third, as population and public health have emerged as key disciplines in the contemporary health ecosystem, more scalable, macro-level, and drill-down studies would inform greater understanding of chronic diseases. Fourth, one would assume that the quality of publicly available data is high and error-free. Lastly, the study is limited to examining associations and correlations and does not investigate causality. Furthermore, we only apply visual analytics and descriptive analytics, which have limitations in and of themselves.

## 5. Implications

This study has analyzed chronic conditions in conjunction with several demographic variables, including gender and race. There are widespread variations in the prevalence of diverse chronic diseases, the number of hospitalizations for specific diseases, and the diagnosis and mortality rates for different states. For some chronic diseases—such as diabetes, arthritis, and obstructive pulmonary —the prevalence in the east is higher than in other regions, while, there is higher prevalence for other conditions, such as asthma, in the northeast. The south and midwest also show their own prevalence of chronic diseases. Likewise, there are variations for hospitalization and mortality rates. In addition, there are gender differences related to chronic conditions. For example, women tend to have higher cases per 100,000 for asthma-related hospitalizations. Men, on the other hand, appear to have higher mortality rates for chronic obstructive pulmonary disease, diabetes, chronic kidney, and others. Also, when we examined chronic conditions by race, we noticed that American Indian or Alaska Natives had higher mortality rates for chronic obstructive pulmonary disease, diabetes, chronic kidney, and so on, followed by Black and non-Hispanic groups.

In addition, the study analyzed demographics of mental health, behavior habits, and preventive health. The associations between behavioral health and chronic conditions and between preventive health and chronic conditions were also analyzed. There is a positive relationship between average female coronary heart disease mortality ratio and average female tobacco use ratio. There is a negative relationship between the average pneumococcal vaccination among diabetes patients and the average diagnosed diabetes ratio among the population. Referring to the relationship between behavioral health and overarching conditions, the study found a negative correlation between age-adjusted prevalence percentage of fair or poor self-rated health status among adults aged ≥18 years and binge drinking adults. The current smoking prevalence and sufficiency of sleep among adults is negatively related. The current lack of health insurance is negatively related to both prevalence of current smoking and that of current smokeless tobacco use. The relationship between obesity and poor self-rated health status is positively related. Similarly, current smoking prevalence has a strong, positive correlation with fair or poor self-rated health status. There are different negative or positive correlations between overarching conditions and chronic conditions. For instance, there is a significant positive relationship between the prevalence of a lack of health insurance and that of diagnosed diabetes. But the relationship between prevalence of a lack of health insurance and prevalence of asthma is negatively related.

Finally, we conducted analyses of the differences among chronic conditions. There are obvious clusters between men and women for asthma, although women tend to have a higher prevalence of asthma than men. Overall, prevalence of asthma is negatively related to the prevalence of diabetes. There is a moderate, positive correlation between prevalence of kidney and diabetes, which is akin to the positive correlation between the prevalence of chronic obstructive pulmonary disease and diabetes, arthritis and asthma, arthritis and chronic obstructive pulmonary disease, and asthma and chronic obstructive pulmonary.

## 6. Conclusions

The study makes multiple essential contributions to chronic disease analysis at the patient/physician and the state levels. At the patient level, analysis of chronic conditions and related behavioral factors allows patients to be proactive in managing their conditions as well as modifying behavioral health. In this day and age, patients are eager to assimilate health information from various sources [37,38]. Being informed allows patients to self-monitor and seek appropriate and timely medical care [39,40], contributing to an ultimate care model that is increasingly personalized.

Similar to patients, physicians too have varying information needs in healthcare that need to be satisfied [41]. To physicians, information on chronic conditions and more importantly, associations between multiple conditions and between categories of healthcare, enable developing personalized treatment plans based on patient-specific profiles that integrate various symptoms with environmental and other health data [42]. Additionally, the array of information increases their ability to guide patients in towards lifestyle medicine (making lifestyle changes in healthy diet, exercise etc.) in the management of chronic diseases [43]. The road from sickness to wellness requires integrated efforts from physicians and patients—physicians can coach and guide the patients but the ultimate cross-over to wellness lies in the patients’ hands.

Whereas most studies on chronic diseases focus on specific chronic diseases and are somewhat limited, this study offers comprehensive analysis over multiple categories of chronic diseases at the state-level. By utilizing visual analytics and descriptive analytics, our study offers methods for gaining insight into the relationships between behavior habits, preventative health and demographics, and chronic conditions. Moreover, this study contributes in terms of the methodology of analytics used in the research. It demonstrates the efficacy of data-driven analytics, which can help make informed decisions on chronic diseases.

Going forward, more theoretical and empirical research is needed. Additional studies can address the relationship between chronic disease conditions and other indicators, such as economic, financial, and social. While chronic disease management has become the focus in modern medicine as our population ages and medical costs continue to rise, research should focus on preventive and mitigating policies. The benefits of prevention and its potential to reduce costs and improve outcomes have received the attention of insurance companies, health care plans, and the U.S. Congress. Healthcare systems are now incentivized to reduce readmissions and physicians are encouraged to meet evidence-based quality measures to provide the best outcomes for patients with chronic disease states.

## Figures and Tables

**Table 1:** Chronic diseases and related indicators.

Category	Sub-Category	Variables (Measure)	Definition
Chronic condition	Diabetes	Diabetes (%)	Prevalence of diagnosed diabetes among adults aged ≥18 years—2012–2014
		Hospital diabetes (number)	Hospitalization with diabetes as diagnosis; 2010 and 2013
		Mortality diabetes (per 100,000)	Mortality rate due to diabetes listed as cause of death, 2010–2014
	Arthritis	Arthritis (%)	Prevalence of arthritis among adults aged ≥18 years; 2013–2014
		Fair or poor health—arthritis (%)	Prevalence of fair or poor health among adults aged ≥18 years with arthritis—2013–2014
		Obesity—arthritis (%)	Prevalence of Arthritis among adults aged ≥18 years who are obese—2013–2014
	Asthma	Asthma (%)	Current asthma prevalence among adults aged ≥18 years, through 2012–2014
		Mortality—asthma (case per 100,000)	Asthma mortality rate through 2010–2014
		Hospital—asthma (case per 100,000)	Hospitalizations for Asthma
	Chronic Kidney Disease	Kidney (%)	Prevalence of chronic kidney disease among adults aged ≥18 years—2012–2014
		Mortality—kidney (case per 100,000)	Mortality with end stage renal disease, through 2010 to 2014
	Chronic Obstructive Pulmonary Disease	Pulmonary (%)	Prevalence of chronic obstructive pulmonary disease among adults aged ≥18 years, through 2012 to 2014
		Hospital—pulmonary (case per 100,000)	Hospitalization for chronic obstructive pulmonary disease as any diagnosis of 2010 and 2013
		Mortality—pulmonary (case per 100,000)	Mortality with chronic obstructive pulmonary disease as underlying cause among adults aged ≥45 years, through 2010 and 2014.
Mental health	Mental health	Mental—women (%)	The crude prevalence rate of at least 14 recent mentally unhealthy days among women aged 18–44 years, through 2012 to 2014
		Postpartum (%)	The crude prevalence rate of Postpartum depressive symptoms in 2011
		Mental (number)	The aged-adjusted mean of recently mentally unhealthy days among adults aged ≥18 years, through 2012 to 2014
Behavioral Habits	Alcohol	Binge drink (%)	Binge drinking prevalence among adults aged ≥18 years, through 2012 to 2014
		Heavy drink (%)	Heavy drinking among adults aged ≥18 years, through 2012 to 2014
	Nutrition, Physical Activity, and Weight Status	Physical activity (%)	No leisure-time physical activity among adults aged ≥18 years, through 2012 to 2014
		Tobacco—smokeless (%)	Current smokeless tobacco use among adults aged ≥18 years, through 2012 to 2014
		Tobacco (%)	Current smoking among adults aged ≥18 years, through 2012 to 2014
		Obesity (%)	Obesity among adults aged ≥18 years, through 2012 to 2014
Preventive health	Pneumococcal vaccination	Pneumonia—smoke (%)	Pneumococcal vaccination among noninstitutionalized adults aged 18–64 years who smoke, through 2012 to 2014
		Pneumonia—heart (%)	Pneumococcal vaccination among noninstitutionalized adults aged 18–64 years with a history of coronary heart disease, through 2012 to 2014
		Pneumonia—asthma (%)	Pneumococcal vaccination among noninstitutionalized adults aged 18–64 years with asthma, through 2012 to 2014
		Pneumonia—diabetes (%)	Pneumococcal vaccination among noninstitutionalized adults aged 18–64 years with diagnosed diabetes, through 2012 to 2014
	Immunization	Influenza—asthma (%)	Influenza vaccination among noninstitutionalized adults aged 18–64 years with asthma, through 2012 to 2014;
		Influenza—diabetes (%)	Influenza vaccination among noninstitutionalized adults aged 18–64 years with diagnosed diabetes, through 2012 to 2014
		Influenza—heart (%)	Influenza vaccination among noninstitutionalized adults aged 18–64 years with a history of coronary heart disease or stroke
		Influenza (%)	Influenza vaccination among noninstitutionalized adults aged ≥18 years, through 2012 to 2014
	Smoke	Quit (number)	Quit attempts in the past year among current smokers, through 2012 to 2014
Demographics	Gender	Gender (character)	Male and female
	Ethnicity	Race (character)	Race
Location	State location	Location (character)	50 states and District of Columbia, Guam, Puerto Rico, Virgin Islands
Overarching Conditions	Overarching Conditions	Insurance (%)	Current lack of health insurance among adults aged 18–64 years, through 2012 to 2014
		Poor—self rate (%)	Fair or poor self-rated health status among adults aged ≥18 years
		Sleep (%)	Prevalence of sufficient sleep among adults aged ≥18 years
